# Type A personality, hostility, time urgency and unintentional injuries among Chinese undergraduates: a matched case–control study

**DOI:** 10.1186/1471-2458-13-1066

**Published:** 2013-11-12

**Authors:** Hongying Shi, Xinjun Yang, Jingjing Wang, Haiyang Xi, Chenping Huang, Jincai He, Maoping Chu, Guihua Zhuang

**Affiliations:** 1 Department of Epidemiology and Biostatistics, School of Public Health, Xi’an Jiaotong University, Xi’an, Shaanxi 710061, China; 2 Department of Preventive Medicine, School of Environmental Science and Public Health, Wenzhou Medical University, Wenzhou 325035, China; 3 Department of Psychology, School of Environmental Science and Public Health, Wenzhou Medical University, Wenzhou 325035, China; 4 The First Affiliated Hospital of Wenzhou Medical University, No.2, Fu Xue Road, Wenzhou 325000, China

## Abstract

**Background:**

Associations between type A behaviour pattern (TABP) and injuries are inconsistent. These inconsistencies may be due to different effects of various components of TABP, namely time urgency/impatience, hostility and competitive drive. It is important to examine the relationship between the global TABP, its two components, and unintentional injuries, among undergraduates in China.

**Methods:**

On the basis of a previous cross-sectional study, we conducted a matched case–control study. 253 cases and an equal number of age-, gender-, and major-matched controls were included. The questionnaire solicited socio-demographic information, the experience of injuries, the scale of TABP, and other potential confounding factors. Besides the correlation between the global TABP and injuries, the influences of the two components of TABP on injuries were also evaluated. Conditional logistic regression was used to determine the crude odds ratios (ORs) and adjusted ORs of injury events.

**Results:**

A dose–response relationship was apparent among students who rated themselves higher on the TABP scale (*P-*value for trend, 0.002), with a crude OR of 2.93 (95% CI: 0.93–9.19) for injuries comparing those with TABP to those with type B behaviour pattern (TBBP). After adjustment for potential confounding factors, TABP remained statistically significant, and the adjusted OR was 5.52 (95% CI: 1.43–21.27); from a comparison of students with TABP to those with TBBP. A dose–response relationship was also apparent between the hostility component and nonfatal injuries, both in crude analysis and after adjusting for other confounders. The relationship between time-hurry and injuries was not statistically significant, based on univariate and multivariate analyses.

**Conclusions:**

Both the global TABP and the hostility component were associated with a dose response increase in the risk of non-fatal unintentional injuries among Chinese undergraduates. Further studies need to be conducted to confirm or reject this correlation.

## Background

Type A behaviour pattern (TABP), characterized by time urgency, impatience, and hostility, has been traditionally reported to be associated with coronary heart disease since the 1950s [1,2]. Since that time, there has been a debate on whether TABP is also associated with driving behaviours or injuries. To date, data concerning the relationship between TABP and injuries are inconsistent.

Several epidemiological studies have provided evidence of an association between type A personality and injuries such as road traffic accidents (RTA) [3-8], fall [9], and other injuries [10-12]. A study by Perry in the early 1980s showed that subjects exhibiting more Type A behaviour tended to be more impatient, reported being involved in more accidents, and received more tickets for driving violations than those scoring lower on the Type A scale [5]. In the 1990s, another study demonstrated that runners with high scores on the type A behaviour screening questionnaire experienced significantly more injuries, especially multiple injuries [11]. Nabi’s famous prospective cohort study of the GAZEL cohort in 2005 showed that the risk for serious road traffic accidents (RTAs) increased proportionally with TABP scores: hazard ratios were 1.29 (95% confidence interval (95% CI): 1.03-1.63) for intermediate-level scores and 1.48 (95% CI: 1.16-1.90) for high-level scores relative to low TABP scores after adjustment for potential confounders [3]. However, a review showed that from numerous psychological attributes, only competitive anxiety has been shown to be associated with sports injuries; a personality profile typical of the “injury-prone” athlete does not exist [13]. Other studies also did not find any significant relationship between individual personality and injuries [14-16].

The first key explanation for these inconsistent results is that almost all of the previous literature focused solely on the relationship between global TABP and injuries, and did not evaluate the relative importance of its underlying components [3,6,8-11], namely, the time urgency/impatience component or the hostility component. There has been evidence showing the relationship between subscales of type A personality and diseases [17-19]; especially between hostility and diseases [18-23]. As for the influence of TABP components on injuries, few studies have been conducted, and the results are inconsistent [4,5,7,13,20,24-27]. Another important reason for these inconsistent results may be that some of the previous studies suffered from low sample sizes or did not include important potential confounders such as socio-economic status, smoking and drinking habits [4,5,8,11,25,27].

In our previous cross-sectional study, we also found a relationship between overall TABP and non-fatal injuries; and the adjusted odds ratio (OR) was 2.99 (95% CI: 1.45 –6.14) comparing students with TABP to students with type B behaviour pattern (TBBP) [28]. It remains unclear, however, whether there is an association between the hostility component of TABP and injuries, or between the time urgency/impatience component of TABP and injuries; and if so, whether the association is independent of other traditional risk factors. To further investigate the possible relationship of injuries with type A personality and its subscales, we conducted this matched case–control study in Wenzhou, China. We sought to determine (1) whether global TABP is associated with non-fatal unintentional injuries among undergraduates; (2) whether time urgency, or hostility, are associated with non-fatal injuries among this population; (3) whether these relationships are independent of other risk factors.

## Methods

### Study subjects

A matched case–control study was performed at three universities in Wenzhou, China. Details of the subject selection are published elsewhere [28]. Briefly speaking, we conducted a self-administered questionnaire survey among college students in 2009. The sampling framework was all classes in these three universities, and the participants were selected by a multi-stage random sampling method. The questionnaire solicited socio-demographic information, experience of injuries during the preceding 12 months, and the scale of type A behaviour pattern. Among the selected 2350 students, 97.3% (n = 2287) provided valid replies.

For this study, the case group was selected from students who had reported being injured in the previous cross-sectional study [28]. And the injury outcome was measured by the question “have you been injured during the preceding 12 months? ①Yes ②no”; an injury case was defined as an injury meeting at least one of the following criteria during the year before the survey [29]: (1) an injury for which the student received medical treatment at the school nurse’s office, or received medical care from a doctor at a hospital or a private medical office; (2) an injury for which the student received first aid from his/her schoolmates, teachers, or parents; or (3) an injury that was not treated but caused the student to miss a half day or more of school or regular activities. We excluded students who reported being injured but were not treated at all due to the injury.

Control subjects were from the source population of these case students. That is, for each case patient, a control subject of the same gender, academic major, and age (±1 year) was selected at random from the students who were also surveyed but not injured during the same year. The selection process was performed using SAS macro.

As for the sample size, the number of pairs needed in this study was estimated using the following formula: $M=\frac{m}{\left(p_{0}q_{1}+p_{1}q_{0}\right)}$[30], where $m=\frac{{\left[\frac{u_{\alpha }}{2}+u_{\beta }\sqrt{p\left(1-p\right)}\right]}^{2}}{{\left(p-0.5\right)}^{2}}$; *m* represents the discordant pairs (b + c); *p* = *OR*/(1 + *OR*); *p*_0_ and *p*_1_ represent the proportion of the exposure factor in case and control groups respectively; *p*_1_ = (*p*_0_ × *OR*)/[1 + *p*_0_(*OR* − 1)]; *q*_0_ = 1 − *p*_0_; *q*_1_ = 1 − *p*_1_. According to previous findings, we assumed *p*_0_ = 20%, OR = 2.0 [3], the number of pairs needed was 226.

This survey was conducted in compliance with the Helsinki Declaration, and was reviewed and approved by the Wenzhou Medical University Ethics Committee. Principals of selected schools signed written consent forms. Students’ verbal consents were obtained before the study. During the survey, assurance was given that all questionnaire information about the students would be confidential and only used for research.

### Assessment of hostility and time urgency

The TABP scale revised by the Chinese National Collaborative Study Group for TABP & coronary heart disease [31] was used for all undergraduates, to assess behaviour pattern. This scale is a revised version of some foreign scales such as the scale of Jenkins Activity Survey (JAS), and has been commonly used in China since the 1980s. It has good reliability (Cronbach’s α coefficient is 0.78 in this study). There are 60 items in the scale of TABP, including three dimensions: TU (time urgency), CH (competitive hostility, for simplicity, we used the hostility component in the following text), and L (lie). If the score for the L dimension was higher than 7, the questionnaire was invalid and would be deleted in the final data analysis. Next, we obtained the total score for the scale. The higher the total score on the scale, the closer to the TABP; otherwise, the lower the total score, the closer to the TBBP. According to the mean (mean = 27) and standard deviation(SD = 8) of the total score of the scale, we divided the students into five groups, similar to the previous researchers: A(≥36), mA (28–35), M (27), mB (19–26), and B(≤18) [31], that is, if the total score on the scale is larger than the mean + standard deviation (SD), the student would be grouped into A; if the total score on the scale was less than mean-SD, then the student would be grouped into B; which was a little different from the standard in some other countries [3]. Groups A and mA were combined into one group: type A, and the others were combined into another group: not type A [32].

In order to assess the influences of the two components of the TABP scale on injuries in this study, a total hostility score and a total time urgency score were also used and categorized into approximate quintiles of the distribution. For both hostility and time urgency, higher scores indicated a higher tendency for a particular trait. Both the continuous and categorical versions of these variables were analyzed in relation to the risk of nonfatal injury. We assessed the internal consistency, reliability of the time urgency dimension, and the hostility dimension using Cronbach’s α coefficient. Cronbach’s α coefficient for the TU subscale was 0.64, and Cronbach’s α coefficient for CH was 0.65 in this matched case–control study.

### Assessment of traditional injury risk factors

Because risk factors associated with unintentional nonfatal injuries include individual characteristics and family environment characteristics, we obtained this information by questionnaire in our survey [33].

At the individual level, we included students’ age, gender, body mass index (BMI, weight [kg]/[height (m)]^2^), cigarette smoking (one or more cigarettes per day and smoking for more than half a year), drinking habit (one time per day and 3 months or more), sports (do you like sports?), club activity (have you ever joined any club activity?), study time per day (<4 h, 4-7 h, 8-12 h, >12 h).

At the family level, we surveyed annual per-capita income (<2000, 2000–9999, 10000–29999, 30000–49999, ≥ 50000 yuan), the number of siblings (0, 1 or more), parents’ education and occupation, birthplace (urban, town, rural).

These traditional factors were included in the analyses to control for potential confounding and/or to assess the independent effect of hostility, time urgency, and type A personality on injuries.

### Statistical analysis

First, we examined the distributions of basic characteristics according to case–control status. Secondly, we examined the distributions of these factors according to the different levels of the TABP scale. Then, to examine the association between TABP and non-fatal injuries, conditional logistic regression was carried out to estimate the odds ratios (ORs) and the 95% confidence intervals (CIs). We fit a series of models adjusting for each covariate to assess potential confounding or mediating effects, and a full model adjusting for all covariates as in other studies [17].

In a secondary set of analyses, the relationships of the components of TABP with injuries were analyzed.

To be robust, in all conditional logistic regression models, we initially analyzed the global TABP score, CH score and TU score as numerical variables respectively, then we divided the TU and CH scores into five distinct levels using percentiles (scores below the 20th percentile represented the low level, those ranging between the 20th and 40th percentiles represented the second lowest level, those ranging between the 40th and 60th percentiles represented the intermediate level, those ranging between the 60th and 80th percentiles represented the second highest level, and those above the 80th percentile represented the highest level).

## Results

In the sample of 2287 students who completed valid questionnaire responses in the previously published study, there were 320 injured students; of these, 253 students were drawn and individually matched with controls, 67 were excluded because they were not treated due to the injury, which resulted in 506 individuals in this matched case–control study. Of all the 506 students selected in this case–control study, 15 (3.0%) were type A, 107 (21.1%) were type mA, 31 (6.1%) were type M, 242 (47.8%) were type mB, and 111 (21.9%) were type B. The mean global TABP score of these 506 students was 23.45 (SD = 6.06, range 7–40). The time urgency component had a mean of 10.89 (SD = 3.64, range 3–21), and the hostility component had a mean of 12.56 (SD = 3.43, range 3–23).

Among the 253 cases, 29.6% were type A students; while the proportion was 18.6% in the control group, the McNemar test showed that there was a statistically significant difference between cases and controls, *P* = 0.005. The total score of the TABP scale was also higher in the case group (24.11 ± 6.28), than that of the control group (22.79 ± 5.77), *t* = 2.59, *P* = 0.01. In addition, the mean CH score of cases (12.97 ± 3.58) was also higher than that of control students (12.15 ± 3.23), *t* = 2.86, *P* = 0.005; while the mean TH score of cases (11.14 ± 3.72) was not significantly higher that of control students (10.64 ± 3.55), *t* = 1.57, *P* = 0.12.

### 1 Sociodemographic characteristics of cases and controls

The distributions of the basic characteristics among cases were similar with controls, except for sports and drinking habits between cases and controls (Table 1). There was no statistically significant difference of parental vocations and education levels between these two groups (data not shown).

**Table 1:** Sociodemographic characteristics of injury cases and controls, 2012

**Characteristic**	**Cases (n = 253)**		**Controls (n = 253)**		Test statistic ( *P *-value)^*^
Quantitative or ordinal variables					
Age (year, mean ± SD)	19.6 ± 1.17		19.6 ± 1.15		0.53 (0.594)
BMI (kg/m^2^, mean ± SD)	20.3 ± 2.56		20.2 ± 2.41		0.33 (0.744)
Annual per-capita income, Yuan					0.77 (0.442)
<2000	30 (11.9%)		30 (11.9%)		
2000 ~ 10000	59 (23.3%)		73 (28.9%)		
10000 ~ 30000	83 (32.8%)		71 (28.1%)		
30000 ~ 50000	36 (14.2%)		41 (16.2%)		
> 50000	45 (17.8%)		38 (15.0%)		
Dichotomous variables					
	Exposure in pair^†^				
	Both positive	Only in case	Only in control	Both negative	
No. of siblings	44	62	59	88	0.514
Smoke	0	4	12	237	0.057
Drink	0	4	14	235	0.027
Sports	125	61	35	32	0.009
Club activity	96	69	48	40	0.054

*: *P* value from paired samples *t* test (age, BMI), signed rank sum test (income), McNemar test (dichotomous variables).

^†^: Number of matched pairs in analysis is 253.

### 2 Type A behaviour pattern and sociodemographic variables

In Table 2, we see that there were differences in sociodemographic factors between five different behaviour pattern groups, although most of these differences were not statistically significant. However, a stronger sense of TABP was clearly associated with only-child (*P* =0.027), and drinking habit (*P* =0.017).

**Table 2:** Sociodemographic characteristics by behavior pattern, 2012

**Characteristic**	**Behavior pattern**					**Test statistic**
	**B(n = 111)**	**mB(n = 242)**	**M(n = 31)**	**mA(n = 107)**	**A(n = 15)**	( *P *-value)^*^
Age (year)	19.60 ± 1.06	19.66 ± 1.14	19.81 ± 1.33	19.48 ± 1.20	19.80 ± 1.42	0.77(0.545)
BM I(kg/m^2^)	20.21 ± 1.88	20.39 ± 2.74	21.18 ± 3.12	19.91 ± 2.18	19.69 ± 2.12	1.99(0.094)
Gender (male, %)	43(38.7)	129(53.3)	17(54.8)	49(45.8)	8(53.3)	7.42(0.115)
Major (medicine, %)	57(51.4)	156(64.5)	19(61.3)	74(69.2)	8(53.3)	8.78(0.067)
Sports (yes, %)	72(64.9)	174(71.9)	20(64.5)	71(66.4)	9(60.0)	2.93(0.570)
Club activities (yes, %)	65(58.6)	157(64.9)	20(64.5)	58(54.2)	9(60.0)	4.05(0.399)
Only child (yes, %)	57(51.4)	91(37.6)	7(22.6)	47(43.9)	7(46.7)	10.95(0.027)
Smoke (yes, %)	2(1.8)	7(2.9)	1(3.2)	5(4.7)	1(6.7)	- (0.492)
Drink (yes, %)	1(0.9)	11(4.5)	0(0.0)	3(2.8)	3(20.0)	- (0.017)
Annual per-capita income, Yuan						- (0.895)
<2000	12(10.8)	27(11.2)	6(19.4)	12(11.2)	3(20.0)	
2000 ~ 10000	32(28.8)	62(25.6)	9(29.0)	27(25.2)	2(13.3)	
10000 ~ 30000	31(27.9)	77(31.8)	7(22.6)	32(29.9)	7(46.7)	
30000 ~ 50000	16(14.4)	37(15.3)	5(16.1)	19(17.8)	0(0.00)	
> 50000	20(18.0)	39(16.1)	4(12.9)	17(15.9)	3(20.0)	

*. *P* value from *F* test (age, BMI), Chi_squared test (gender, major, sports, club activities, only child), Fisher’s exact test (smoke, drink, and income).

### 3 Type A behaviour pattern and non-fatal unintentional injuries among undergraduates

First, we found that, compared with the non-type A individuals, the crude OR for unintentional injury among type A subjects was 1.85 (95% CI: 1.21-2.82), *P* = 0.004. After controlling for the number of siblings, family income status, drinking habits, and sports, we got similar results; the adjusted OR was 1.86 (95% CI: 1.20-2.88), *P* = 0.004. When we adjusted further for all variables in Table 2, the adjusted OR was 2.04 (95% CI: 1.29-3.24).

Secondly, we used the global TABP score as an independent variable, fitted the conditional logistic regression model, and found that it was also statistically significant (Table 3); which indicated that there was some association between the TABP and unintentional injuries. When we adjusted for those potential confounding variables, the association was not changed. Table 3 presents the ORs and 95% CIs for developing injuries.

**Table 3:** Odds ratios (95% CI) for unintentional injuries among Chinese undergraduates according to the global TABP scale

**Model controlling for:**	**Global TABP score**		OR (95% CI) for TABP category^*^					
	**OR (95% CI)**	***P***	**B (111)**	**mB (242)**	**M (31)**	**mA (107)**	**A (15)**	***P***
-	1.041(1.009 ~ 1.075)	0.011	1	1.28(0.81 ~ 2.04)	1.55(0.68 ~ 3.51)	2.21(1.25 ~ 3.92)	2.93(0.93 ~ 9.19)	0.045
Sports	1.040(1.008 ~ 1.074)	0.014	1	1.21(0.75 ~ 1.93)	1.58(0.69 ~ 3.62)	2.11(1.18 ~ 3.76)	3.06(0.95 ~ 9.86)	0.049
No. of siblings	1.042(1.009 ~ 1.075)	0.011	1	1.29(0.81 ~ 2.06)	1.56(0.69 ~ 3.53)	2.22(1.25 ~ 3.92)	2.91(0.93 ~ 9.13)	0.045
Income	1.041(1.009 ~ 1.075)	0.011	1	1.29(0.81 ~ 2.05)	1.55(0.68 ~ 3.51)	2.21(1.25 ~ 3.91)	2.96(0.94 ~ 9.33)	0.044
Drink	1.042(1.009 ~ 1.075)	0.011	1	1.35(0.84 ~ 2.86)	1.42(0.62 ~ 3.24)	2.21(1.24 ~ 3.93)	4.03(1.18 ~ 13.83)	0.035
Model^†^	1.042(1.009 ~ 1.076)	0.012	1	1.30(0.80 ~ 2.12)	1.50(0.65 ~ 3.48)	2.13(1.18 ~ 3.87)	4.28(1.24 ~ 14.74)	0.040
Model^#^	1.053(1.018 ~ 1.090)	0.003	1	1.52(0.91 ~ 2.55)	1.61(0.67 ~ 3.87)	2.67(1.44 ~ 4.96)	5.52(1.43 ~ 21.27)	0.012

*: According to the total score of the TABP scale, we divided the students into five groups: A (≥36), mA (28 ~ 35), M (27), mB (19 ~ 26), and B (≤18).

^†^: A model adjusting for sports, only child, income, and drinking habits.

#: A full model adjusting for sports, only child, income, drinking habits, smoking habits, BMI, club activity.

**Figure 1:**
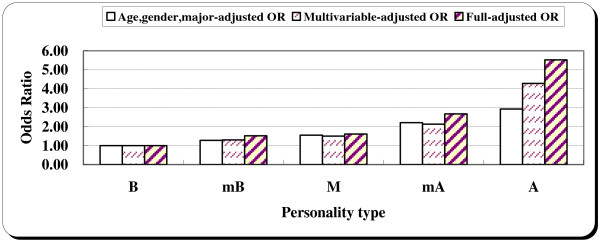
Age, gender, major-adjusted, multivariable-adjusted and fully-adjusted odds ratios for unintentional injuries by personality type.

We then used the TABP category (type B, mB, M, mA, A) as the independent variable; ORs increased with TABP categories. A dose response relationship was obviously observed, and adjusting for the other factors did not change the association (Table 3). In the multivariable models controlling for the number of siblings, family income status, drinking habits, and sports, the adjusted OR for non-fatal injuries among those with type A personality was 4.28 (95% CI: 1.24–14.74), while the adjusted OR among those with mA was 2.13 (95% CI: 1.18–3.87), both compared with the type B individuals (Table 3, Figure 1). In the fully-adjusted model controlling for the number of siblings, family income status, drinking habits, sports, smoking habit, BMI, club activity; the adjusted OR increased slightly (Table 3, Figure 1).

### 4 Hostility and non-fatal unintentional injuries among undergraduates

First, we used the hostility component (CH) score as the continuous independent variable, and found that both the crude OR and the adjusted OR were about 1.08 (Table 4).

**Table 4:** Odds ratios (95% CI) for unintentional injuries among Chinese undergraduates according to the CH component

**Model controlling for:**	**CH score**		**OR (95% CI) for CH category according to quintile**					
	**OR (95% CI)**	***P***	**Quintile 1**	**Quintile 2**	**Quintile 3**	**Quintile 4**	**Quintile 5**	***P***
			**≤9(107)**	**10-12(143)**	**13(60)**	**14-16(121)**	**17 + (75)**	
-	1.082(1.024 ~ 1.144)	0.005	1	0.82(0.49 ~ 1.37)	1.19(0.63 ~ 2.24)	1.51(0.89 ~ 2.59)	2.54(1.32 ~ 4.91)	0.006
Sports	1.080(1.021 ~ 1.143)	0.007	1	0.77(0.46 ~ 1.29)	1.25(0.65 ~ 2.37)	1.43(0.83 ~ 2.47)	2.52(1.29 ~ 4.91)	0.006
No. of siblings	1.082(1.023 ~ 1.144)	0.006	1	0.82(0.50 ~ 1.36)	1.19(0.63 ~ 2.24)	1.51(0.89 ~ 2.59)	2.55(1.32 ~ 4.95)	0.007
Income	1.080(1.021 ~ 1.142)	0.007	1	0.81(0.49 ~ 1.35)	1.17(0.62 ~ 2.21)	1.49(0.87 ~ 2.56)	2.47(1.28 ~ 4.79)	0.007
Drink	1.080(1.021 ~ 1.143)	0.007	1	0.82(0.49 ~ 1.37)	1.24(0.65 ~ 2.34)	1.50(0.87 ~ 2.57)	2.56(1.31 ~ 4.98)	0.007
Model^*^	1.073(1.013 ~ 1.137)	0.016	1	0.75(0.44 ~ 1.26)	1.28(0.67 ~ 2.45)	1.38(0.80 ~ 2.40)	2.40(1.21 ~ 4.76)	0.009
Model^#^	1.090(1.026 ~ 1.158)	0.005	1	0.86(0.50 ~ 1.49)	1.48(0.75 ~ 2.93)	1.59(0.89 ~ 2.81)	2.90(1.42 ~ 5.93)	0.006

*: A model adjusting for sports, only child, income, and drinking habits.

#: A full model adjusting for sports, only child, income, drinking habits, smoking habits, BMI, club activity.

Then we used the quintiles of CH scores as the independent variable. In crude matched-pair analysis, the measure of CH was associated with a dose–response increase in the risk of non-fatal injuries (*P*-value for linear trend, 0.006). The OR for unintentional injuries among those with the highest rating of CH was 2.54 (95% CI: 1.32-4.91), compared to those with the lowest rating of CH.

**Figure 2:**
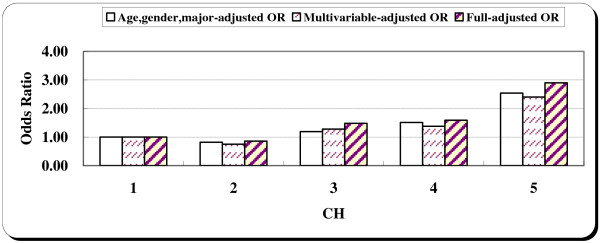
Age, gender, major-adjusted, multivariable-adjusted and fully-adjusted odds ratios for unintentional injuries by CH component quintiles.

In multivariable models controlling for age, gender and major by design and drinking habits, family income status, sports and number of siblings, the measure of competitive drive and hostility remained significantly associated with unintentional injuries among undergraduates (Table 4). The adjusted OR for non-fatal injuries among those with a CH score larger than 17 was 2.40 (95% CI: 1.21–4.76), compared to those with CH score less than 9 (Table 4, Figure 2). In the fully-adjusted model controlling for the number of siblings, family income status, drinking habits, sports, smoking habits, BMI, and club activity, the adjusted OR increased slightly.

### 5 Time urgency and non-fatal unintentional injuries among undergraduates

**Figure 3:**
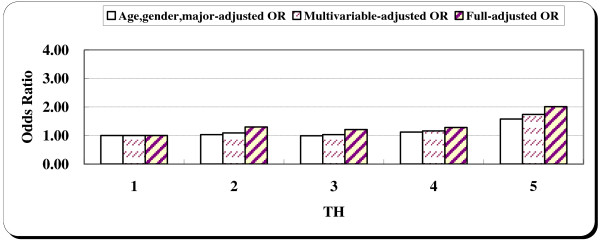
Age, gender, major-adjusted, multivariable-adjusted and fully-adjusted odds ratios for unintentional injuries by TU component quintiles.

The same process of analysis was applied for the time urgency (TU) component; the results are presented in Table 5. Surprisingly, there were no statistical associations between time urgency and injuries in both the crude model and the adjusted models controlling for other confounders. The dose response relationship between the time urgency component and injuries is also not statistically significant (Table 5, Figure 3).

**Table 5:** Odds ratios (95% CI) for unintentional injuries among Chinese undergraduates according to the TU component

**Model controlling for:**	**TU score**		**OR (95% CI) for TU category according to quintile**					
	**OR(95% CI)**	***P***	**Quintile 1**	**Quintile 2**	**Quintile 3**	**Quintile 4**	**Quintile 5**	***P***
			**≤8(149)**	**9-10(93)**	**11-12(102)**	**13-14(79)**	**15 + (83)**	
-	1.040(0.990 ~ 1.093)	0.119	1	1.03(0.60 ~ 1.76)	0.99(0.59 ~ 1.67)	1.12(0.64 ~ 1.96)	1.58(0.89 ~ 2.81)	0.571
Sports	1.040(0.989 ~ 1.093)	0.126	1	1.03(0.59 ~ 1.77)	0.98(0.58 ~ 1.67)	1.11(0.63 ~ 1.95)	1.56(0.88 ~ 2.79)	0.602
No. of siblings	1.042(0.991 ~ 1.096)	0.106	1	1.04(0.60 ~ 1.79)	1.00(0.59 ~ 1.70)	1.14(0.64 ~ 2.02)	1.60(0.90 ~ 2.85)	0.557
Income	1.043(0.992 ~ 1.096)	0.097	1	1.01(0.59 ~ 1.74)	0.99(0.59 ~ 1.67)	1.12(0.64 ~ 1.97)	1.62(0.91 ~ 2.88)	0.520
Drink	1.043(0.992 ~ 1.097)	0.099	1	1.09(0.63 ~ 1.88)	1.01(0.59 ~ 1.72)	1.11(0.63 ~ 1.95)	1.66(0.93 ~ 2.97)	0.520
Model^*^	1.052(0.999 ~ 1.108)	0.057	1	1.09(0.62 ~ 1.92)	1.03(0.60 ~ 1.77)	1.16(0.64 ~ 2.08)	1.74(0.96 ~ 3.16)	0.456
Model^†^	1.053(1.000 ~ 1.110)	0.052	1	1.30(0.72 ~ 2.35)	1.21(0.69 ~ 2.15)	1.28(0.70 ~ 2.36)	2.01(1.08 ~ 3.76)	0.302

*: A model adjusting for sports, only child, income, and drinking habits.

^†^: A full model adjusting for sports, only child, income, drinking habits, smoking habits, BMI, club activity.

Finally, we analyzed the association between hostility and injuries. This time we also adjusted for the time urgency component. In this model, hostility remained independently associated with non-fatal injuries among undergraduates, and the dose–response relationship was also evident (Compared with the first group, ORs and 95%CI for the other four groups were 0.82(0.47-1.43), 1.42(0.71-2.87), 1.50(0.83-2.72), 2.64(1.21-5.75) respectively, *P* = 0.022).

## Discussion

Human factors, especially psychological characteristics of individuals, appear to be the most important contributing factors of injuries. This matched case–control study demonstrated a robust association between type A behaviour pattern and the risk of unintentional injuries among undergraduates in Wenzhou, China. These data also indicated a robust association between hostility and injuries, but no statistically significant association between sense of time urgency and injuries. These associations were independent of age, gender, academic major, sports, family income status, number of siblings, drinking habits, smoking habits, club activities, and body mass index. The association between hostility and injuries appeared to be independent of the other component-time urgency, suggesting that hostility is indeed an independent predictor of increased risk of unintentional injuries. In addition, when we used the TABP and hostility variables as numerical or ordinal measures, the results did not change.

This confirmed previous suspicions that perhaps only a subset of Type A individuals are at higher risk for injuries, or, perhaps the type A personality construct is only important as it pertains to hostility and anger [19]. A 1974 study showed that 13 per cent of these reports described injuries due to aggressive behaviour [20]. Another study showed that a sub-group of Type A individuals who were disliked by their co-workers had significantly higher risks for injury than type A individuals who were liked, and all Type B individuals [24]. A study in China also found that aggressiveness was a risk factor for unintentional injuries [25]. ORs for aggressive behaviours were elevated across all categories of suicide behaviours including serious suicidal thoughts, specific suicidal plans, and suicidal attempts [26]. A latest case–control study on the association of TABP with accident proneness among drivers showed that the difference of the time urgency component was statistically significant between case drivers and controls, while the difference of the hostility component was not significant [4]; which seems different from our findings. However, the sample size of this study was only 46 pairs.

Hostility may affect injuries through one or more pathways. Hostile individuals display heightened physiological reactivity in some situations, report greater degrees of interpersonal conflict and less social support, and may have more unhealthy daily habits, and therefore may be at increased risk for subsequent coronary heart disease [23,34,35], and other life-threatening illnesses [18,36-38].

The present study has several strengths. We used a college-based matched case–control study, which increased the statistical power of the study, in comparison with previous cross-sectional studies. The cases in this study were randomly selected from all injured cases found in the previous cross-sectional study, and the characteristics between the selected individuals and all injured individuals were similar, which indicated that the case patients in this study were representative. The control students were randomly selected from the source population of cases, and were matched with cases by gender, age and academic major; therefore, the comparability between cases and controls is good. Third, this was a college-based, not a hospital-based study, which avoided selection bias such as the Berkson bias in hospital-based case–control studies; while increasing the cooperation of subjects, and the accuracy of the data. Hence, this provides an avenue for further research using a college-based case–control study.

The study also has some important limitations. As with any case–control study, psychosocial factors such as type A personality and hostility may be differentially recalled among cases and controls. However, the subjects were unaware of our hypothesis, and the hostility hypothesis is not widely known to the lay public; therefore, we believe that recall bias is an unlikely explanation for our findings. Additionally, as this is not a prospective study, it is not possible to conclude that the personality type preceded the injury. Second, there may be some residual confounding factors as is usually the case in all other observational epidemiologic studies. A review showed that type A behaviour in women is positively correlated with socioeconomic status, occupation, and education [39]. Therefore, it is important to assess the relationship between type A personality and injuries after adjusting for the influence of confounding factors. In our study, we adjusted for the influence of age, gender, academic major in medical school, sports (do you like sports?), family income status, number of siblings, and drinking habits. We assumed that students who like sports are likely to engage in sports more frequently; therefore, we did not collect detailed information of frequency, intensity of sports, or the types of sports. There must be some differences between liking sports and the actual frequency of sports, which should be taken into account in future research. We also did not include other potential confounding factors such as driving while not wearing helmets [40-42], risky use of cellular phones while driving [3,43], insufficient sleep [44,45], associated training [46], drug use, caffeine use, and diagnosis of conduct disorder [47] which would influence the likelihood of injury, especially road traffic accidents, and may be associated with personality. However, some of these factors such as driving, drug use, and caffeine use are not very common among these undergraduate students; and should therefore not influence the main results of this study. Finally, our results only apply to non-fatal unintentional injuries among undergraduates, which limits its generalizability. The population of this study is college students who will soon graduate, which makes it relatively difficult to implement interventions or conduct a prospective study. Considering that there are many private car drivers in Wenzhou city, we would conduct further research such as cohort studies or intervention studies among this population to confirm previous findings.

## Conclusions

In summary, our case–control study found a positive association between type A behaviour pattern and unintentional injuries, especially the hostility component, and unintentional injuries among undergraduates in Wenzhou, China. Therefore, students who display hostility should be alert to their own personality, and should take measures to adjust their emotions, using stress-reduction methods such as regular exercise. Public health professionals and experts on injury prevention and control should pay more attention to type A behaviour individuals, especially those who have hostility characteristics, during the process of health education and health promotion; and apply individualized interventions.

## Competing interests

The authors declare that they have no competing interests.

## Authors’ contributions

HYS conceptualized the study, performed the analysis, and wrote the first draft of the article. GHZ revised the manuscript and made valuable suggestions. XJY, CPH participated in the design of the study. JCH provided valuable suggestions on psychological evaluation. MPC participated in coordination. JJW and HYX participated in acquisition of data and edited the paper. All authors have read and approved the final manuscript.

## Pre-publication history

The pre-publication history for this paper can be accessed here:

http://www.biomedcentral.com/1471-2458/13/1066/prepub
